# Optimal housing temperatures for mice to mimic the thermal environment of humans: An experimental study

**DOI:** 10.1016/j.molmet.2017.10.009

**Published:** 2017-10-31

**Authors:** Alexander W. Fischer, Barbara Cannon, Jan Nedergaard

**Affiliations:** 1 Department of Molecular Biosciences, The Wenner-Gren Institute, The Arrhenius Laboratories F3, Stockholm University, SE-106 91 Stockholm, Sweden; 2 Department of Biochemistry and Molecular Cell Biology, University Medical Center Hamburg-Eppendorf, DE-20246 Hamburg, Germany

**Keywords:** Ambient temperature, Basal metabolic rate, Human, Lower critical temperature, Mouse, Thermoneutral, Thermoregulation, BMR, basal metabolic rate, EE, energy expenditure, LCT, lower critical temperature, RMR, resting metabolic rate, RQ, respiratory quotient

## Abstract

**Objectives:**

The laboratory mouse is presently the most common model for examining mechanisms of human physiology and disease. Housing temperatures can have a large impact on the outcome of such experiments and on their translatability to the human situation. Humans usually create for themselves a thermoneutral environment without cold stress, while laboratory mice under standard conditions (≈20° C) are under constant cold stress. In a well-cited, theoretical paper by Speakman and Keijer in Molecular Metabolism, it was argued that housing mice under close to standard conditions is the optimal way of modeling the human metabolic situation. This tenet was mainly based on the observation that humans usually display average metabolic rates of about 1.6 times basal metabolic rate. The extra heat thereby produced would also be expected to lead to a shift in the ‘lower critical temperature’ towards lower temperatures.

**Methods:**

To examine these tenets experimentally, we performed high time-resolution indirect calorimetry at different environmental temperatures on mice acclimated to different housing temperatures.

**Results:**

Based on the high time-resolution calorimetry analysis, we found that mice already under thermoneutral conditions display mean diurnal energy expenditure rates 1.8 times higher than basal metabolism, remarkably closely resembling the human situation. At any temperature below thermoneutrality, mice metabolism therefore exceeds the human equivalent: Mice under standard conditions display energy expenditure 3.1 times basal metabolism. The discrepancy to previous conclusions is probably attributable to earlier limitations in establishing true mouse basal metabolic rate, due to low time resolution. We also found that the fact that mean energy expenditure exceeds resting metabolic rate does not move the apparent thermoneutral zone (the lower critical temperature) downwards.

**Conclusions:**

We show that housing mice at thermoneutrality is an advantageous step towards aligning mouse energy metabolism to human energy metabolism.

## Highlights

• High time-resolution indirect calorimetry reveals true resting metabolic rate in mice.
• Mice at thermoneutrality display average energy expenditure 1.8 times their RMR (BMR).
• This relationship very closely resembles the human situation.
• Lower critical temperature is not influenced by enhanced energy expenditure.
• To model human metabolism, 30 °C remains the optimal housing temperature for mice.

## Graphical abstract

**Figure:**
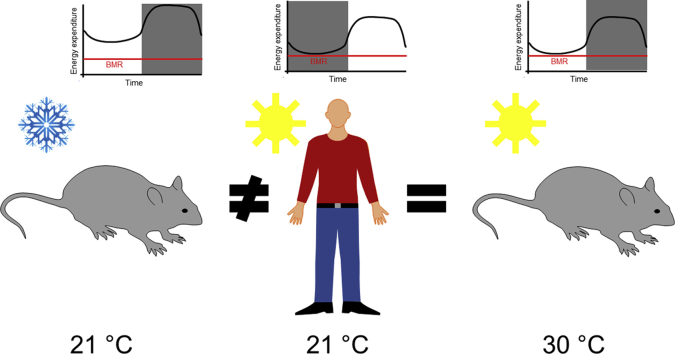

## 1 Introduction

In metabolic research, there is an increasing understanding that the environmental temperature may dramatically affect the outcome of experiments. This is particularly the case for mice. Mice have a thermoneutral zone encompassing some degrees around 30 °C but are normally kept under housing conditions around 20 °C. They therefore constantly need to produce extra heat to defend their body temperature, and this means that they constantly have to maintain a substantially increased metabolism as compared to that at thermoneutrality; this affects many metabolic parameters.

Indeed, a large series of recent contributions have experimentally and theoretically demonstrated the profound influence of these thermal conditions on various important parameters (e.g. [1], [2], [3], [4], [5], [6], [7], [8], [9], [10], [11], [12], [13], [14], [15], [16], [17], [18]). From these studies, the implications would be that housing mice at thermoneutrality would make mice better models for humans, in health and disease. However, in a well-cited paper in Molecular Metabolism, this notion was challenged by Speakman and Keijer [19]. These authors imply from data in the literature that close to standard housing conditions (≈20° C) are metabolically superior to thermoneutral housing for mimicking the human situation.

It is evidently of utmost importance for medically related research to ensure that experimental conditions in mice resemble human conditions. We have therefore here experimentally examined the tenets of Speakman and Keijer. Speakman and Keijer put forward two main arguments supporting housing under standard conditions. One argument is based on the observation that humans under normal life conditions display energy expenditure values of around 1.6–1.8 times basal metabolic rate (BMR) [19], [20], [21]. Therefore, the authors suggest that mice should be housed under conditions where metabolism is increased 1.6-fold over basal metabolism; i.e. they should be below thermoneutrality.

Secondly, the authors argue that normal activity generates heat above resting metabolic rates and that, as a consequence, humans (and implicitly all mammals) would prefer exposure to temperatures below thermoneutrality in order to dissipate the extra heat.

In the present investigation, we have attempted to experimentally study these assumptions. We particularly addressed the issue that metabolic rates in mice are often examined in experimental set-ups with many parallel cages, leading to low time resolution, as the metabolism of each cage/mouse is then only sampled for 1 min every 15–60 min. The outcome will thus in reality represent a mean metabolic rate which may include bouts of physical activity or other change in energy expenditure. In contrast, human BMR is assessed under strict conditions of physical inactivity. To address this discrepancy, we employed high time-resolution calorimetry. This technique enabled us to identify the true BMR in mice.

## 2 Methods

### 2.1 Animals

Male C57BL/6NCrl mice were purchased at 12 weeks of age from Nova (Sweden). Mice were single-caged and housed at room temperatures of 30 °C, 21 °C, or 4 °C (4 °C after a one-week acclimation period at 18 °C) with a 12/12 h light–dark cycle. The mice were supplied with a cardboard house and wood-wool nesting material. The mice had free access to water and were fed a chow diet (Lactamin R70) ad libitum. After 4 weeks of acclimation to the respective temperature, the mice were placed in metabolic chambers and analyzed as described below. All experiments were approved by the Animal Ethics Committee of the North Stockholm Region.

### 2.2 Calorimetric measurements

High time-resolution energy expenditure (EE) was measured in an indirect calorimetry system (INCA, Somedic, Hörby, Sweden). The mice – in their home cages but without house and nesting material – were transferred to a sealed chamber (5.6 l) with a temperature controller to maintain a stable, adjustable temperature. After calibration of the oxygen sensors, oxygen consumption and carbon dioxide production were measured for one minute every other minute. During the other minute, the incoming air was measured. The mice were transferred into the chambers the day before the measurement to allow them to acclimate to the chambers. The measurements were performed at the respective acclimation temperature (however, 10 °C for the cold-acclimated mice), and the mice acclimated to 21 °C and 4 °C were additionally analyzed at 30 °C. The mice had free access to food and water and were on a 12 h/12 h light/dark cycle in the chambers.

### 2.3 Basal and resting metabolic rates

According to the Glossary of Terms for Thermal Physiology [22], basal metabolic rate (BMR) is defined from oxygen consumption/energy expenditure measurements determined in a rested awake organism, fasted sufficiently long to be in a post-absorptive state and being in a thermoneutral temperature zone. Resting metabolic rate (RMR) is the same as BMR (i.e. at rest and at thermoneutrality) but the animal cannot be guaranteed to be in a post-absorptive state [22]. As discussed earlier by Speakman [23], [24], it may be preferable, therefore, to always use the term RMR when the absorptive status is unclear. We have followed this suggestion here, although we allow ourselves to use the term RMR as specified to a defined ambient temperature, indicated as a subscript (e.g. RMR_30_ for RMR determined at 30 °C), in extension of a suggestion by Speakman [23], [24].

### 2.4 Metabolic calculations

Energy expenditure (EE) in watts [W] was calculated using a modified Weir equation [25]:

$$\text{Energy expenditure}\;\left[\text{W}\right]\;=\;(\text{0}\text{.}2716\frac{\text{W×min}}{\text{ml}}\times \text{V}{\left({\text{O}}_{2}\right)}_{\text{consumed}}\left[\frac{\text{ml}}{\text{min}}\right]\text{)}\;+\;\text{(0}\text{.}07616\frac{\text{W×min}}{\text{ml}}\times \text{V}{\left({\text{CO}}_{2}\right)}_{\text{produced}}\left[\frac{\text{ml}}{\text{min}}\right]\text{)}$$

True BMR (post-absorptive) can hardly ever be measured in mice since the prolonged fasting necessary to achieve a post-absorptive state will lead to major behavioral alterations. We therefore calculated day- and night-time RMR. Since mice are mostly asleep during day-time and not only at rest (the definition of BMR states that the organism should be awake [22]), and as sleep metabolic rates are lower than “resting-but-awake” metabolic rates, day-time mouse RMR may be lower than the “true” BMR. Correspondingly, the night-time RMR may overestimate the “true” BMR of the mice, since a fully post-absorptive state will not be reached.

**Figure 1:**
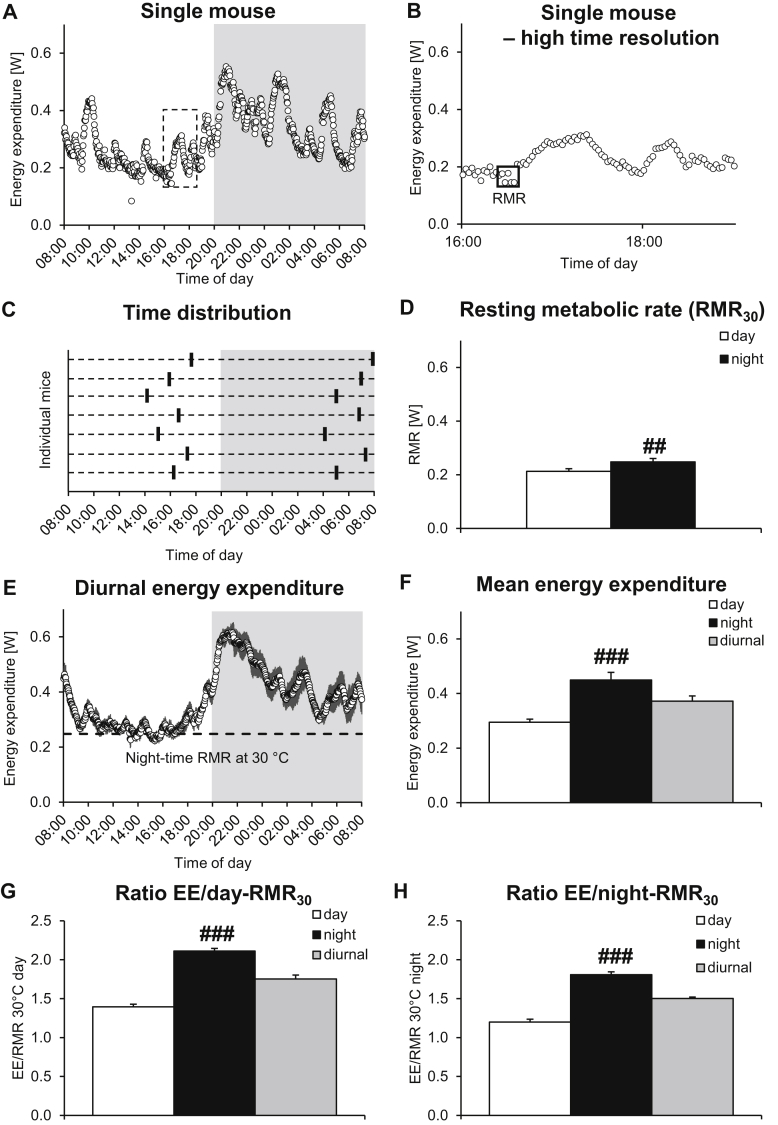
Establishment of the ratio between resting metabolic rate and average energy expenditure in mice at thermoneutrality reveals similarity to human metabolism. To examine the relationship between resting and total metabolism in mice under thermoneutral conditions, male 12-week-old C57BL/6 mice were acclimated to 30 °C for 4 weeks and energy expenditure was recorded with high time-resolution. **A.** Representative 24 h energy expenditure trace of a single mouse showing phases of higher and lower energy expenditure during both day- and night-time. Gray background indicates dark phase. Box indicates the time-frame shown in the high time-resolution figure in B. **B.** High time-resolution of a 3 h interval of the representative trace from A showing phases of higher and lower energy expenditure. The lowest 5 consecutive values are indicated and their average was defined as RMR (Box). Note that there were lower single values, but the box represents the lowest 10 min and these were the values that were used for further analysis. **C.** Time distribution of the lowest 10 min of energy expenditure used for calculation of the resting metabolic rate for the individual mice. Each stippled line represents one mouse. Note the clustering in the end of the dark phase and shortly before the end of the light phase. **D**. Average resting metabolic rate for day- and night-time. **E.** Mean diurnal energy expenditure trace of 7 mice. Dashed line indicates the calculated average resting metabolic rate (RMR) during night-time from D. **F.** Average day, night, and diurnal energy expenditure. **G**. Ratio between day-time, night-time and diurnal energy expenditure and day-time RMR_30_. **H**. Ratio between day-time, night-time and diurnal energy expenditure and night-time RMR_30_. All values in D–H are the means ± SEM of 7 mice. ## and ### indicates p ≤ 0.01 and 0.001 between day- and night-time by Student's paired t-test (for each mouse); for the group, there was no significant difference between the mean day- and night-time RMR_30_ (panel D).

The resting metabolic rate (RMR) at each ambient temperature was defined as the average of the 5 consecutive energy expenditure values that represented the 10 min with the lowest energy expenditure (see Figure 1B), during the day or the night. The mean energy expenditure (EE) was calculated as the average day or night energy expenditure (or the diurnal energy expenditure). The ratio between mean EE and RMR was calculated separately for day and night energy expenditure, as well as for diurnal energy expenditure.

For the Scholander-like plots (energy expenditure vs. temperature plots), average energy expenditure was plotted against the corresponding environmental temperature. Note that each temperature represents individual groups of mice and this is thus not exactly in accordance with the original Scholander setup, in which individual animals are gradually exposed to different temperatures, being exposed to each temperature for only a couple of hours. However, the strategy used here enabled us to clearly separate resting and active phases during the 24 h measurements.

For calculation of the lower critical temperature (LCT), metabolism at 30 °C was considered to be within the thermoneutral zone. LCT was thus calculated from the linear regression curve of the 21 °C and 10 °C energy expenditure vs. temperature as follows:

$$\text{LCT}\left[{}^{\circ}\text{C}\right]=\frac{{\text{EE}}_{30\;{}^{\circ}\text{C}}\left[\text{W}\right]-\left(y-{\text{intercept}}_{21\;{}^{\circ}\text{C}-10\;{}^{\circ}\text{C}}\left[\text{W}\right]\right)}{{\text{slope}}_{21\;{}^{\circ}\text{C}-10\;{}^{\circ}\text{C}}\left[\frac{\text{W}}{{}^{\circ}\text{C}}\right]}$$

Since different mice were used for each temperature, only the average LCT could be calculated.

### 2.5 Thermal preference test

Male 15-week-old C57BL/6JRj mice acclimated to 21 °C were used for the thermal preference experiment. A detailed description of these experiments can be found in [26]. Briefly, an aluminum channel (800 mm × 100 mm × 100 mm) covered by a Plexiglas lid was used. One end of the gradient was placed in a warm water bath (55 °C), the other end in a cold water bath (21 °C). The gradient temperature was measured every 5 cm of the gradient. To examine the temperature preference, a mouse was placed in the gradient and video-recorded. A tracking software was used to calculate the coordinates of the mouse within the gradient. The coordinates were used to calculate the corresponding temperatures. For analysis, the probability to stay within each predefined 2 °C-areas during 1.5 h after 30 min adaptation to the gradient was calculated for each mouse. The probability is the percentage of total time spent within each 2 °C segment. Additionally, the preferred ambient temperature during the sleeping phase was examined by recording the temperature zone in which the mice were sleeping at the end of the 2 h experiment.

### 2.6 Statistics

Analyses were performed using Microsoft Excel. Statistical significance was examined using unpaired (between mice) or paired (within mice) Student's t test; p ≤ 0.05 was considered to be a statistically significant difference. All data shown are mean ± SEM.

## 3 Results

### 3.1 Establishment of the ratio between resting metabolic rate and average energy expenditure in mice at thermoneutrality reveals similarity to human metabolism

In order to establish the ratio between RMR and average energy expenditure for mice, we performed high time-resolution indirect calorimetry in a thermoneutral environment (30 °C) using mice acclimated to 30 °C for 4 weeks. As expected, and as seen for a single mouse in Figure 1A, the mice displayed a diurnal rhythmicity in energy expenditure, with day-time levels representing the resting/sleeping phase of the animal. Even within this resting phase, bouts of higher energy expenditure could be observed, followed by phases of low energy expenditure. A similar pattern could also be seen during the night-time, while overall energy expenditure was clearly higher during the dark phase.

In Figure 1B, we show high time-resolution energy expenditure during the end of the light phase (indicated by the stippled box in Figure 1A). In this representation, the high but consistent variation in metabolic rate is prominent, and it is clear that, if low time-resolution calorimetry would have been used, a value would have been obtained that included phases of both lower and higher metabolic rates. In low time-resolution calorimetry, this value would generally be considered the RMR at thermoneutrality. Instead, we defined the resting metabolic rate (RMR_30_) of the mice as the average value of the 5 consecutive energy expenditure measures representing the 10 min with the lowest energy expenditure (Box) during the entire day phase. In Figure 1C, we have plotted the time distribution pattern of the identified RMR period (that shown in Figure 1B) for each mouse. There was a clear clustering of this RMR period at the end of the day phase; this indicates that the occurrence of these time periods with low metabolic rates were not chance events but represented true metabolic events.

This way of defining the resting metabolic rate was also applied to the night phase, where RMR also clustered at the end of the phase (Figure 1C). In Figure 1D, we show the mean day- and night-time RMR. Although the night-time RMR_30_ was significantly higher than the day-time RMR_30_ when calculated for each mouse, the inter-mice variation was such that there was not a significant difference between the day- and night-time RMR mean values.

In Figure 1E, we show the average diurnal pattern of energy expenditure for all the mice. The pattern was remarkably similar in the different mice, as indicated by the low standard error, indicating rather synchronized alterations between phases of higher and lower energy expenditure, only observable with high time-resolution calorimetry. As seen in Figure 1F, mean energy expenditure was – as expected – significantly higher during night-time as compared to the day.

Figure 1G,H shows the ratio between energy expenditure and day-time RMR_30_ (Figure 1G) or night-time RMR_30_ (Figure 1H). Based on the calculation using the day-time RMR_30_ (Figure 1G), the mean energy expenditure during day-time, representing the resting phase of the animal, was 1.4 times higher than RMR. During the night phase, representing the active phase of the animal, the EE/RMR_30_ ratio was 2.1. The ratio between diurnal 24 h energy expenditure and day-time RMR_30_ was 1.8. Remarkably, this value very closely resembles the values calculated for humans based on 24 h energy expenditure measurements using doubly-labeled water methods [20], [21] versus directly measured BMR. It thus appears that under thermoneutral conditions, mouse metabolic rate is strikingly similar to human metabolism, in that the daily average metabolism is about two thirds higher than the BMR (RMR). Similar conclusions would be reached when using night-time RMR_30_ for the calculations (Figure 1H).

### 3.2 In mice housed under standard conditions, energy expenditure does not mimic human conditions

**Figure 2:**
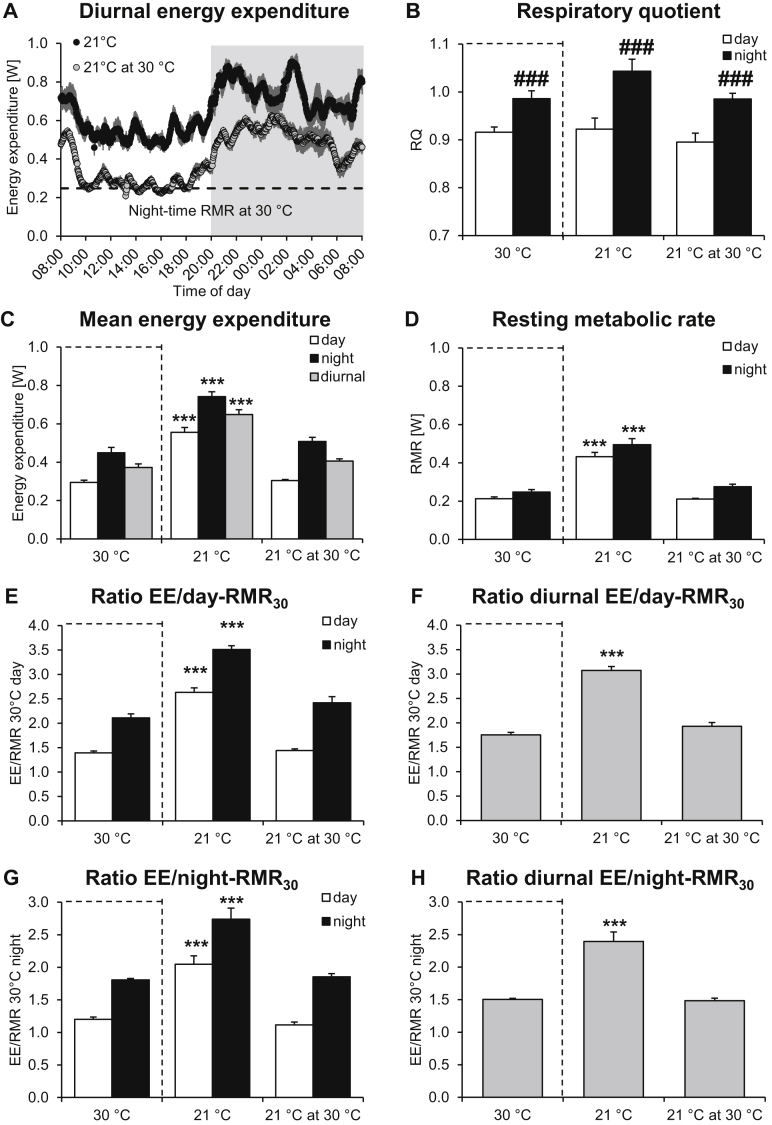
In mice housed under standard animal house conditions, energy expenditure does not mimic human conditions. To examine the relationship between resting and total metabolism in mice under standard animal housing conditions, male 12-week-old C57BL/6 mice were acclimated to 21 °C for 4 weeks and energy expenditure was recorded. **A.** Average energy expenditure trace for 7 mice. The mice were first measured at 21 °C and subsequently at 30 °C. Dashed line indicates the calculated average resting metabolic rate during night-time in the 30 °C-acclimated mice from Figure 1. **B.** Average respiratory quotient showing typical diurnal rhythmicity. Note that here and in the following graphs, the 30 °C values from Figure 1 are shown for comparison (indicated by dashed boxes). **C.** Mean day-time, night-time, and diurnal energy expenditure. **D.** Average day-time and night-time RMR at different temperatures. The RMR_30_, i.e. the RMR of mice housed under standard conditions (21 °C) but measured acutely at 30 °C (“21 °C at 30 °C”) was used to calculate the ratios shown in E–H. **E, F**. Ratio between day-time (E), night-time (E) and diurnal (F) energy expenditure and day-time RMR_30_. **G, H**. Ratio between day-time (G), night-time (G) and diurnal (H) energy expenditure and night-time RMR_30_. All values are the means ± SEM of 7 mice. In B, ### indicates p ≤ 0.001 between day- and night-time by Student's paired t-test (for each mouse). Note that statistically significant day–night differences are not indicated in the other graphs. *** indicates p ≤ 0.001 for the values different from those of 30 °C-acclimated mice.

Standard animal facilities often operate at a housing temperature of 21 °C. Speakman and Keijer proposed that temperatures close to such standard housing conditions would mimic human metabolic conditions better than thermoneutrality [19]. We therefore analyzed the metabolic rates of mice housed and measured under such standard conditions. As seen in Figure 2A (upper trace), also at 21 °C, the mice showed a clear diurnal pattern of energy expenditure, with night-time levels being higher than day-time. In order to examine possible sustained effects of acclimation to a colder temperature on RMR measured at thermoneutrality, we also measured energy expenditure of the same mice when they were acutely transferred to thermoneutral conditions. As seen in Figure 2A (lower trace), the metabolic rates of these mice under thermoneutral conditions were only about half of the rates measured in the same mice at 21 °C.

The mice displayed the expected diurnal rhythm in respiratory quotient (RQ) (Figure 2B). For reasons of comparison, the values of the mice acclimated to and measured at 30 °C (from Figure 1) are also shown here and in the following graphs (in dashed boxes).

In agreement with the traces shown in Figure 2A, calculated energy expenditure rates at 21 °C were significantly higher than at 30 °C (Figure 2C). Diurnal energy expenditure rates were essentially almost doubled at 21 °C, undoubtedly causing large alterations in many aspects of metabolism. Examination of the energy expenditure at 30 °C of the mice acclimated to 21 °C (“21 °C at 30 °C”) did not reveal energy expenditure data different from those of the 30 °C-acclimated mice.

Figure 2D shows day- and night-time RMR at the different temperatures, as defined in Figure 1B. Clearly, when exposed to normal housing conditions (21 °C), the animals also demonstrated periods of rest, but metabolism never decreased to the levels observed in the mice acclimated to thermoneutrality (30 °C). However, when these mice housed under standard conditions were examined at 30 °C (“21 °C at 30 °C”), the RMR_30_, just as total energy expenditure, was unaffected by the acclimation temperature and not different from the RMR_30_ of mice acclimated to and measured at thermoneutrality.

Since Speakman and Keijer [19] suggested that it is necessary to expose mice to standard housing conditions (≈20° C) to be able to reach a ratio between daily energy expenditure and RMR of 1.5–1.7 and thus to mimic the human situation, we calculated these ratios also under standard conditions. To do so, the ratios between the energy expenditure values obtained at 21 °C (middle columns in Figure 2C) and the RMR_30_ determined in these mice at 30 °C (right columns in Figure 2D) were obtained. As seen in Figure 2E, the observed ratio at 21 °C profoundly exceeded the human ratio of 1.6, being 2.6 at day-time and 3.5 at night-time. Diurnal energy expenditure was on average 3.1 times RMR_30_ (Figure 2F). Thus, the ratio between the metabolism of mice under standard housing conditions and their RMR clearly does not mimic the ratio between energy expenditure and BMR observed in humans under normal living conditions. Similar conclusions were obtained using night-time RMR_30_ of these mice (Figure 2GH) or the RMR_30_ obtained (Figure 1D) in mice acclimated to and measured at thermoneutrality (not shown)

### 3.3 No effect on lower critical temperature of mean energy expenditure being higher than resting metabolic rate

The lower critical temperature (LCT) is the environmental temperature below which a mammal needs to increase its metabolism in order to counteract heat loss. It has been implied that the fact that mean energy expenditure is higher than resting metabolic rate would change the apparent lower critical temperature and thus the (apparent) thermoneutral zone [19]. In such an interpretation, the extra heat produced would allow the organism to stay euthermic in colder environments without having to further increase metabolism; this would mean that keeping the mice at temperatures somewhat below the thermoneutral zone would not increase the mean metabolic rate.

**Figure 3:**
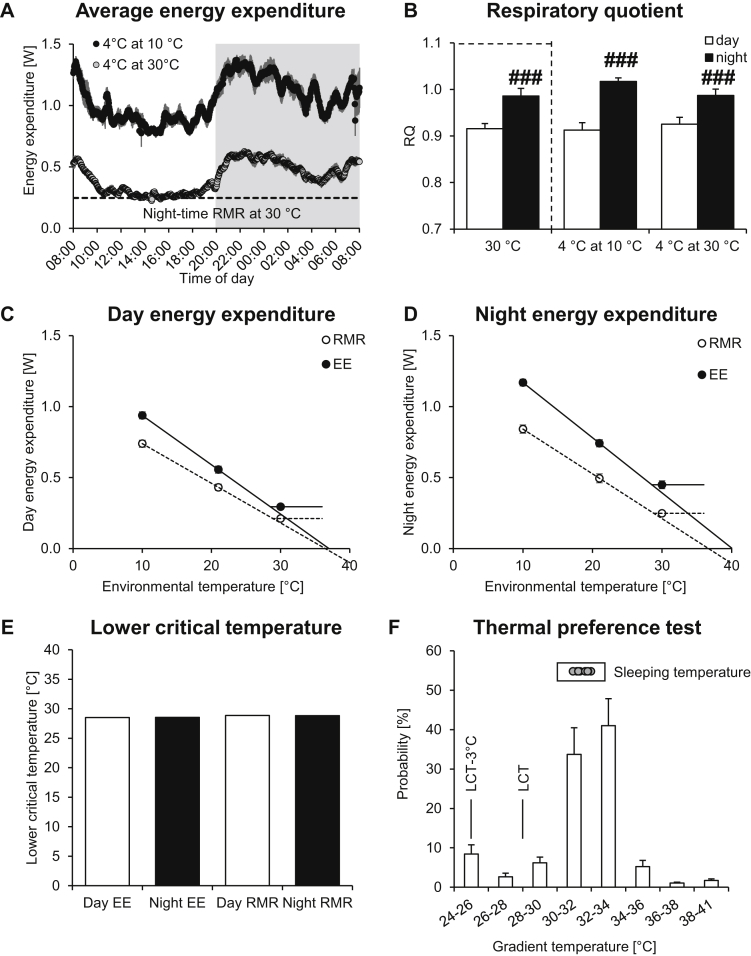
Determination of the lower critical temperature. **A.** Male 12-week-old C57BL/6 mice were acclimated to 4 °C (after 1 week at 18 °C) for 4 weeks, and energy expenditure was recorded at 10 °C and 30 °C. Average energy expenditure trace for 7 mice. The mice were first measured at 10 °C and subsequently at 30 °C. Dashed line indicates the calculated average resting metabolic rate (RMR_30_) during night-time in 30 °C-acclimated mice from Figure 1. **B.** Average respiratory quotient showing typical diurnal rhythmicity. Note that the 30 °C values already presented in Figure 1 are shown for comparison (indicated by dashed box). **C, D**. Scholander-like plot for day-time (C) and night-time (D) RMR and total EE. Resting metabolism, as well as average energy expenditure, were plotted against the environmental temperature in a Scholander-like plot to examine the effect on lower critical temperature of activity, as compared to inactive resting. Note that the data from 21 °C and 30 °C are from the calculations presented in Figure 1, Figure 2. The horizontal line indicates the metabolism within the thermoneutral zone (30 °C), the regression lines of the 21 °C and 10 °C energy expenditure represent the increased need to produce heat in order to maintain euthermia. The intercept of the 2 lines indicates the lower critical temperature. The regression lines extrapolate to the apparent defended body temperature. **E.** Calculated lower critical temperature for the different conditions displayed in C and D. Note that different groups of mice were used at each temperature; thus, no individual lower critical temperature values for each mouse could be calculated. **F.** Thermal preference test. Probability distribution of staying in the indicated temperature zones of a metal temperature gradient is shown, as well as the preferred sleeping temperature (dots). Note that the distribution, but not the sleeping temperature, has already been shown in [26]. It should be noted that 24 °C was the temperature at the end of the gradient box, where the mice initially seemed to seek protection when put into the gradient. LCT indicates the lower critical temperature from E; LCT – 3 °C indicates the preferred temperature as suggested by Speakman and Keijer [19]. All values are the means ± SEM of 7 mice for A–C and 9 mice for F. Note that in C and D, the SEM was generally smaller than the size of the symbols.

Within the limitations of the data available, we tested this tenet in mice. We used the energy expenditure values obtained in the experiments in Figure 1, Figure 2 (mice measured at 30 °C and 21 °C, respectively), as well as data from a group of mice acclimated to the cold and measured at 10 °C. As seen in Figure 3A, the metabolism of cold-acclimated mice at 10 °C (upper trace) was more than 3 times higher than that measured in the same mice at thermoneutrality (lower trace), while the diurnal rhythmicity in energy expenditure (Figure 3A) and RQ (Figure 3B) was observed at both temperatures.

To perform a Scholander-like analysis [27], we plotted the resting metabolic rates for the different temperatures against the corresponding environmental temperature (open circles in Figure 3CD, for day and night energy expenditure) and calculated the lower critical temperature. It was ≈28 °C during both day-time and night-time (Figure 3E). We then also plotted the mean energy expenditure data versus the different ambient temperatures (filled circles in Figure 3CD). As seen, despite the fact that the mean energy expenditure values were evidently higher than the RMR at 30 °C, the Scholander analysis of the mean energy expenditure data do not indicate a left-shift of the lower critical temperature. In contrast, it is clear that the mean energy expenditure also increases below a critical temperature that is calculated to also be ≈28 °C (Figure 3E). Thus, the fact that mean energy expenditure is higher than resting energy expenditure (RMR) is not empirically reflected in the apparent thermoneutral zone being broader, although this indeed is what could be expected from theoretical considerations.

The x-axis-intercept of the Scholander curve is considered to be a measure of the (apparent) defended body temperature of the animal. The use of either the RMR or the energy expenditure data did not affect the apparent defended body temperature during day-time (≈37 °C), while during night-time, the apparent defended body temperature was higher (≈40 °C) when calculated based on total energy expenditure than when based on RMR. This may be interpreted to indicate that the mice defend a higher body temperature during the active phases than when at rest [28]. Actually, the defended resting temperature, as derived from the RMR at the different ambient temperatures, does not show a day-to-night shift but remains at 37 °C; it would seem that it is only during the periods of activity that a higher body temperature is defended during the night.

The increase in energy expenditure with decreasing environmental temperature represents the minimal extra heat needed by the organism to compensate for the increase in heat loss and thus to maintain euthermia. The slope of this curve is a function of the animal's insulation, since higher insulation will reduce the heat loss, thereby decreasing the need to perform thermogenesis to maintain a stable body temperature [19], [27], [29]. As is evident in Figure 3C,D, the slope during activity was higher, representing lower apparent insulation of the animals. This difference in insulation may be due to changes in piloerection, tail blood flow, and/or a greater surface area exposed to the environment during physical activity.

### 3.4 During day-time, mice do not seek environmental temperatures below thermoneutrality when active

It has been considered that in order to lose heat, humans prefer lower environmental temperatures when active than when at rest; this temperature has been suggested to be 3 °C below the lower critical temperature that is relevant for clothed humans [19]. It has also been suggested that a similar 3 °C-lower-than-critical-temperature behavior would be observed in other mammals, including mice [19]. We examined the existence of this behavioral response in mice. To do so, we re-analyzed an earlier behavioral experiment, in which mice were placed during day-time in a 24–41 °C temperature gradient (detailed description can be found in [26]). The time that the mouse spent within pre-defined temperature zones was recorded during a 90-min experiment (Figure 3F). Note that this part of the data has already been presented in [26]. In this figure, we have further indicated the lower critical temperature (LTC) for the mice, as determined above, i.e. ≈28 °C, as well as LCT minus 3 °C (i.e. ≈25 °C), that would be the suggested preferred temperature according to [19]. As seen in Figure 3F, the mice did not display any preference for the low temperatures during this study. Although the experiment encompassed both periods of activity and inactivity, albeit during day-time only, the mice stayed most of the time in the 30–32 and 32–34 °C zones, clearly above their lower critical temperature. Thus, the preferred temperature of the mice is within the thermoneutral zone irrespective of the mice being active or not. This outcome is in agreement with earlier reported day-time results [8], [30], [31], but see Discussion (section 4.5).

We also analyzed the preferred sleeping temperature of these mice, i.e. the ambient temperature at which the mice were sleeping at the end of the experiment (gray circles). During this phase, the preferred temperature was in the same range as when both active and inactive phases were incorporated. If the mice would systematically choose colder temperatures when active, the distribution pattern should be markedly shifted to the left of the sleeping temperatures. This was not the case.

## 4 Discussion

In the present investigation, we have experimentally examined the tenet proposed by Speakman and Keijer that close to standard housing conditions for mice represent adequate models for human metabolism [19]. The main argument for this proposal is related to observations that human daily energy expenditure exceeds human basal metabolic rate by a factor of around 1.6, and, therefore, that it would be necessary to keep mice below their thermoneutral zone in order to achieve this enhanced metabolic rate in mice. Our experiments do not support this suggestion: we find that already at thermoneutrality, daily energy expenditure of mice is about 1.6 times basal metabolic rate, and any temperature lower than this would impose a higher metabolic burden on the mice.

### 4.1 Mice show rhythmic alterations in energy expenditure

The use of high time-resolution calorimetry clearly unveiled that mice, both during the active and resting phases, show rhythmic and close to synchronized bursts of high energy expenditure (“activity”), even at thermoneutrality (Figure 1, Figure 2, Figure 3A). As the mice are totally isolated from the surroundings during the recordings in metabolic chambers, the bursts cannot be synchronized through sensory interaction between the mice but must originate from internal cues. The background for the bursts is not clear; since they are visible at thermoneutrality they cannot represent an acute recruitment of extra heat production to defend body temperature. However, it is likely that they are associated with the fluctuations in body temperature and physical activity that occur in mice [32] (our unpublished observations). Whereas their function is not clarified here, these bursts markedly affect the measurements of energy expenditure and the ability to establish true resting metabolic rate (RMR). Thus, if standard (low time-resolution) calorimetry is used, the outcome will be a metabolic rate that includes both true RMR and the activity bouts, and such measurements must necessarily result in values for apparent RMR that are higher than the true values. Indeed, if we mimic this from the data collected here, by calculating the apparent RMR as sampled every 16 min instead of every 2 min and taken the mean of the lowest 5 values during the day, we would obtain a 1.2 times higher apparent RMR than that obtained with 2 min resolution. If we sampled every 30 min, it would be as high as 1.4 times the present RMR value. Thus, the RMR obtained in this way would approach the mean EE. The calculated mean energy expenditure would not be affected by the different time resolution (not shown).

### 4.2 Mice at thermoneutrality show metabolic similarity to humans

After having established the true resting metabolic rate through high time-resolution calorimetry, we found that when housed under thermoneutral conditions, mice displayed a daily mean energy expenditure (EE) that was about 1.8 times higher than their true resting metabolic rate (RMR) (Figure 1GH). Remarkably, the EE/RMR ratio obtained is thus practically the same as the ratio observed for humans under normal living conditions [19], [20], [21]. (Humans in whole-room calorimeters may show somewhat lower EE/RMR ratios – but this is not of relevance here, as the assessment refers to using caged mice as metabolic models for “free-living” humans.) We have not performed an analysis here of the nature of the processes that consume the energy above RMR; it should include very little classical nonshivering thermogenesis but would include obligatory and perhaps some facultative diet-induced thermogenesis. The rest should, just as in humans, primarily result from physical activity; indeed, despite the limited area of the cages used, we have observed that, even at thermoneutrality, the mice may move as much as 300 m per day (not shown). This is rather considerable physical activity considering that the body length of the mouse is only 10 cm.

The observation that EE/RMR is ≈1.8 was made possible by the ability to perform high time-resolution indirect calorimetry to identify true resting metabolic rates at thermoneutrality. If low time-resolution is used, what is measured is not BMR (RMR) but is averaged energy expenditure, as can be seen in Figure 1. It is thus correct, as pointed out by Speakman and Keijer [19], that the mean energy expenditure at 21 °C is about 1.8-fold that at 30 °C; thus, if mean energy expenditure at 30 °C is equated with RMR, it would be correct that human-like metabolic conditions would be obtained at 21 °C. However, we show here that the true RMR is much lower and that EE at 30 °C cannot be equated with true RMR (which is in reality the case if data from Scholander plots are used to estimate BMR, as in [19]).

### 4.3 Mice under standard housing conditions are under considerable metabolic stress

As already at thermoneutrality the ratio of energy expenditure to true RMR is equivalent to that observed in humans, exposure of mice to any temperature below thermoneutrality imposes a higher metabolic stress that what is relevant for humans. Thus, according to our data, the mean energy expenditure of mice housed at 21 °C is not 1.6 but about 3 times higher than the true RMR (Figure 2E–H). Thus, undoubtedly, standard laboratory housing conditions impose a metabolic stress on mice that greatly exceeds the relative level of metabolism observed in normal “free-living” humans. Indeed, a constant metabolism three times higher than BMR is equivalent to that induced by keeping naked humans at 5 °C [33] day and night - or to be walking about 100 km per day (estimated based on [34]). Undoubtedly, many metabolic (and other) parameters will be affected by such conditions.

### 4.4 Activity does not affect the lower critical temperature

Concerning the suggested effect of activity on the preferred environmental temperature, the theoretical arguments of Speakman and Keijer [19] would seem persuasive in that the extra heat produced from activity in the thermoneutral zone should mean that the lower critical temperature – the temperature below which the mouse has to increase metabolism to counteract heat loss – should become lower. However, the measurements presented here demonstrate that this is not the case. At temperatures below 28 °C, an increase in both apparent resting metabolic rate and mean energy expenditure is seen (Figure 3CD). Thus, irrespective of whether RMR or EE is considered, temperatures below 28 °C impose an increasing metabolic demand on the mice.

### 4.5 Preferred temperature is largely within the thermoneutral zone

At thermoneutrality, increased activity would result in higher EE (at temperatures below thermoneutrality this is not necessarily the case, as heat from activity would replace the extra heat already being produced to counteract heat loss [35]). Theoretically, this requires that more heat would need to be dissipated and this should therefore lead to the mice preferring an ambient temperature below thermoneutrality. However, our observations did not support this behavioral adjustment: the mice remained within the thermoneutral zone. Probably, changes in heat loss through e.g. altered tail vasoconstriction (cf. e.g. [26]) are sufficient to compensate for the extra heat generated by modest activity. The mice also chose the thermoneutral zone for sleeping, and, when active during the study, they did not prefer to move to a lower temperature. All these observations are in line with earlier observations also made during day-time [8], [30], [31].

However, when such studies were extended to night-time – i.e. the active phase of the mice – Gordon and colleagues did observe that during the early part of the night, when the mice are most active, they showed the expected change in preferred temperature: they chose ≈26 °C [8], [31]. Thus, if exposed to standard animal house temperatures of ≈21 °C, mice are not at their preferred temperature at any time; if exposed to thermoneutral conditions (≈30 °C), the mice are at their preferred temperature about 18 out of 24 h.

### 4.6 Optimal housing conditions

All the data obtained here are based on mice studied singly and without access to nests. It may be suggested – as was done by Speakman and Keijer [19] – that group housing and nest building would substantially affect the behavior and metabolic physiology of the mice, as huddling and nests would diminish heat loss; therefore standard conditions should not affect metabolic parameters as much as implied here. However, although group housing and nest material do affect both preferred temperature and metabolic parameters of the mice, the effects are comparatively small [31], [36].

Thus, the outcome of the present analysis is that mouse metabolic parameters become more similar to those of humans when the mice are kept at thermoneutrality than when they are kept under standard animal house conditions. It may be envisaged that by introducing a daily rhythm in animal house temperature, decreasing the temperature from ≈29 °C to ≈26 °C during the 6 first hours of the night, may further optimize the housing conditions of the mice to move the mice even more towards being metabolic models for humans.

### 4.7 Conclusions

We conclude that thermoneutral temperatures remain the preferred method of modeling human conditions for metabolic research – and probably for many other types of medically related research.
